# Evaluation of the effects of L-carnitine on medaka (*Oryzias latipes*) fatty liver

**DOI:** 10.1038/s41598-017-02924-5

**Published:** 2017-06-05

**Authors:** Koichi Fujisawa, Taro Takami, Aya Matsuzaki, Toshihiko Matsumoto, Naoki Yamamoto, Shuji Terai, Isao Sakaida

**Affiliations:** 1 Center for Regenerative Medicine, Yamaguchi University School of Medicine, Minami Kogushi 1-1-1, Ube Yamaguchi, 755-8505 Japan; 2 Department of Gastroenterology and Hepatology, Yamaguchi University Graduate School of Medicine, Minami Kogushi 1-1-1, Ube Yamaguchi, 755-8505 Japan; 3 Division of Gastroenterology and Hepatology, Graduate School of Medical and Dental Sciences, Niigata University, 1–757 Asahimachidori, Chuo-Ku, Niigata 951–8510 Japan

## Abstract

Lifestyle-related diseases have become a major issue in recent years. The increasing incidence of fatty liver underlines the urgency with which the issues of non-alcoholic fatty liver disease (NAFLD) and non-alcoholic steatohepatitis (NASH) need to be addressed. L-carnitine is a compound known to transport fatty acids into the mitochondria to enhance β-oxidation-mediated metabolism of fats. In this study, the effects of L-carnitine administration on fatty liver of medaka (*Oryzias latipes*) were analysed, to check for disease improvement and metabolic changes. Additionally, the effects of the concomitant administration of L-carnitine and eicosapentaenoic acid (20:5n-3) (EPA) were investigated. Findings indicated reduced lipid deposition, increase in metabolites associated with β-oxidation, and significant reduction in fatty acid levels in the liver, implying improvement in fatty liver condition. Concomitant administration of L-carnitine and EPA resulted in further benefits, via changes in fatty acid composition in the medaka fatty liver model.

## Introduction

L-carnitine, a compound (molecular weight: 161.21 kDa) known to be involved in lipid metabolism, is synthesized from lysine and methionine mainly in the liver, with the involvement of vitamin C, ferrous ions, and niacin. The required amino acids are obtained from the diet and the compound is accumulated mostly in muscles. L-carnitine is administered for the treatment of congenital lipid metabolism disorders, such as carnitine transporter deficiency and carnitine deficiency, caused by acquired conditions such as dialysis. Even otherwise, L-carnitine is widely taken as a supplement by healthy individuals, but its lipid metabolism efficiency under conditions of excessive intake has been questioned^1^.

L-carnitine is taken up by the cells through the action of a carnitine transporter (SLC22A5) located on the cell membrane, as a necessary component inside the cell in the transport of long-chain fatty acids through the mitochondrial membrane. Since the inner mitochondrial membrane is impermeable to acyl-CoA on its own, fatty acyl-CoA on the outer mitochondrial membrane binds to L-carnitine through the action of carnitine acyl transferase I enzyme to temporarily generate fatty acyl carnitine. This complex is capable of passing through the inner membrane via the acyl-carnitine/carnitine transporter, following which the fatty acyl group is translocated from L-carnitine to coenzyme A present within the mitochondria by carnitine acyltransferase II enzyme. This results in the formation of fatty acyl-CoA, which is oxidized by enzymes within the matrix. Free L-carnitine is translocated back to the intermembrane space via the acyl-carnitine/carnitine transporter, available for the binding of a new fatty-acyl-CoA molecular. In this manner, L-carnitine contributes to fat transport and oxidation within the cell^2^.

The number of patients with a fatty liver is increasing and studies with the long-term objective of improving the fatty liver-associated conditions (NAFLD) and (NASH) have been conducted. NAFLD is diagnosed in patients who do not drink alcohol and in whom a fatty liver is identified via histopathology, imaging (ultrasound, computed tomography (CT), or magnetic resonance imaging (MRI)), and in whom symptoms attributable to other potential causes are not detected. NASH is a severe form of NAFLD involving steatohepatitis that is pathologically characterized by macrovesicular lipid deposition, ballooning degeneration of hepatocytes, infiltration of inflammatory cells, and perivenular or pericellular fibrosis around the central venous area (zone 3). Patients with NASH are at risk of developing cirrhosis/liver cancer and new therapies must be developed to combat the illness. The efficacy of L-carnitine administration in the treatment of fatty liver has been reported in humans and mice. Although in some studies on the effects of L-carnitine added in the feed of relatively large edible fish, better fish growth and changes in lipid utilization were reported, no effect was observed in other such studies. Factors including administration period, amino acid intake, dosage, and carnitine palmitoyl transferase (CPT) activity are considered associated with these discrepancies^3, 4^, suggesting that the possible use of the compound in this context is yet to be fully explored.

In recent years, small fish such as medakas and zebrafish, have gained attention as new model organisms. These fish are smaller than rodents such as mice, which reduces required space as well as cost. In addition, methods for preparing transgenic and knockout fish are well established based on the genome project. They also tend to grow and proliferate rapidly, and large-scale screening can easily be performed. Among small fish, especially, the medaka possesses an ability to hibernate, has high prolificacy, grows rapidly, is omnivorous and possesses carbohydrate/lipid metabolism functions similar to those found in mammals^5^.

Of significance to our study, a high-fat diet (HFD)-fed medaka model has been established^6, 7^. It has previously been reported that drug assessment using a HFD-fed medaka model is feasible^8^. However, no studies of the effects of L-carnitine on fatty liver found in this model have been reported. In the present study, where metabolomic analysis and lipid analysis were performed, the effects of L-carnitine administration on fatty acid metamorphosis in the medaka model were first shown. In addition, since eicosapentaenoic acid (EPA) is known to decrease upon administration of L-carnitine, we analysed the change in lipid profile caused by concomitant administration of EPA with L-carnitine, regarding which only a few detailed reports have been found.

## Results

### The effects of L-carnitine on body weight, liver morphology and enzyme profile

**Figure 1:**
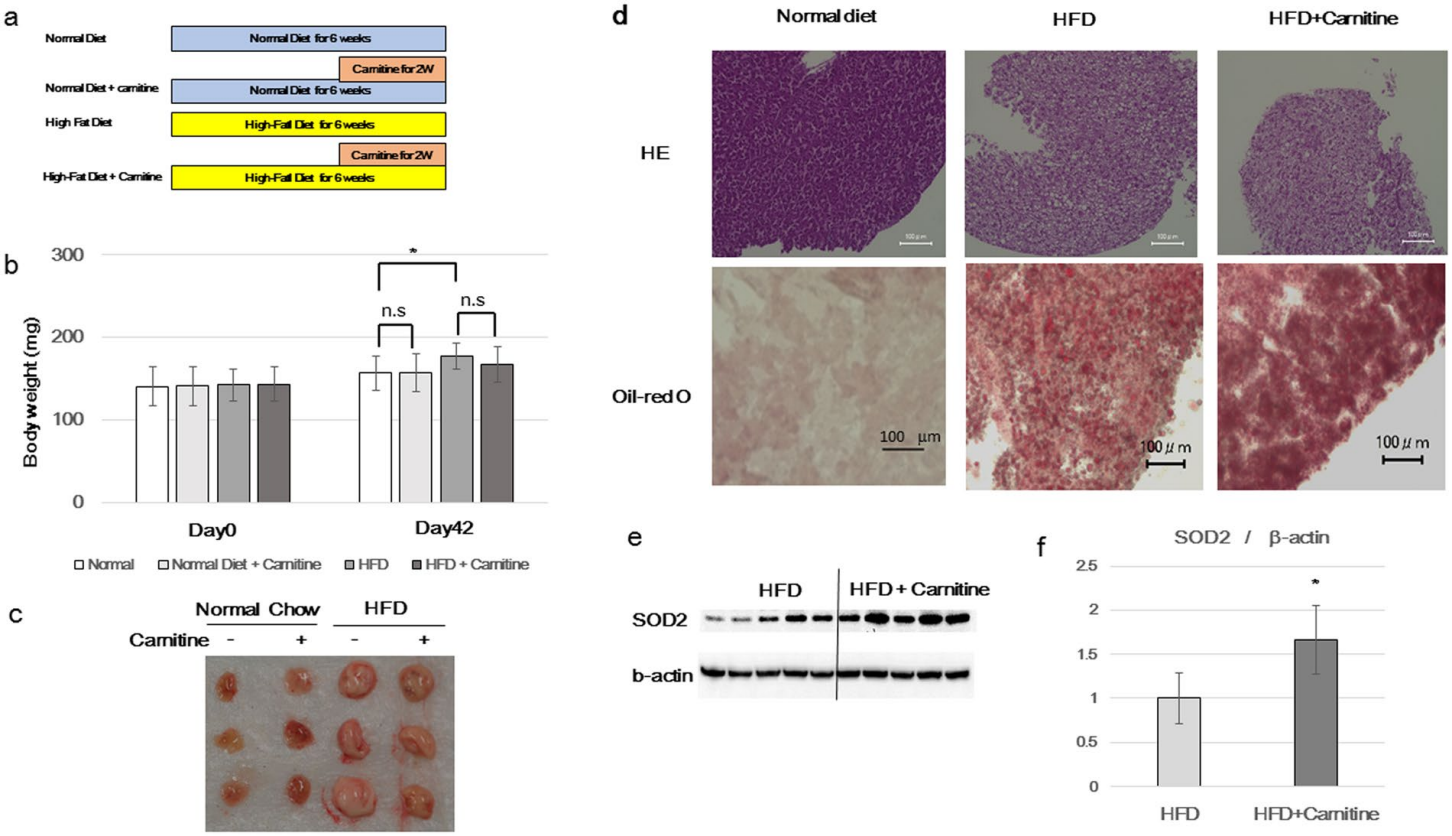
Comparison of body weight, liver size, tissue staining, and protein expression between the HFD group and the HFD + L-carnitine group. (**a**) A schematic diagram of the methods of feeding and agent administration. Medakas (n = 20 in a tank) were fed either a normal diet or HFD and raised for 4 weeks. Following this period, 1 mM L-carnitine was administered, and for 2 weeks the animals were raised and the water was replaced every 3 days until the medakas were sacrificed for analysis. (**b**) Change in body weight following L-carnitine administration. There was no clear difference in body weight among the groups before L-carnitine administration. On Day 42, body weight significantly increased in HFD group compared to normal diet group, but there was no difference between the HFD group and the HFD + L-carnitine group. *Represents p < 0.05 (HFD group compared to normal diet group). n.s represents not significant (normal diet group compared to normal diet + carnitine group, HFD + carnitine group compared to HFD group). (**c**) A photograph of isolated livers on Day 42. The livers in the HFD group are significantly enlarged compared to those in the normal diet group. (**d**) Staining of liver sections on Day 42. Top: Haematoxylin and eosin staining. Bottom: Oil-red O staining. (**e**) The expression analysis of SOD2 by western blotting. The extracted protein samples from the liver in the HFD group (left five lanes) and the HFD + L-carnitine group (right five lanes) were subjected to electrophoresis. Top: anti-SOD2 antibody. Bottom: β-actin antibody (loading control). The corrected signal intensity of SOD2 compared with that of β-actin can be seen in the graph. SOD2 expression is significantly higher in the HFD + L-carnitine group.

Adult medakas were fed either a normal diet or an HFD for a month, followed by additional L-carnitine administration for a further 2 weeks (Fig. 1a). There was a significant increase in body weight in the HFD group compared to that in the normal diet group. No significant change in body weight was observed following L-carnitine administration in either group (Fig. 1b). The isolated medaka livers were whitish and enlarged in the HFD group. Macroscopically, no clear changes were detected following L-carnitine administration in either group (Fig. 1c). The hematoxylin and eosin (H&E) staining of liver sections revealed a greater deposition of lipid droplets in the normal diet group (Fig. 1d upper). Oil-red-O staining of liver sections revealed more lipid accumulation in the HFD group than in the normal diet group. Additionally, a decrease in lipid accumulation was observed following L-carnitine administration to the medaka in the HFD group (Fig. 1d lower). Furthermore, western blot analysis was performed and an increased expression of an antioxidant enzyme, superoxide dismutase 2 (SOD2) following L-carnitine administration, was seen (Fig. 1e).

### Metabolomic analysis by CE-TOF-MS

**Figure 2:**
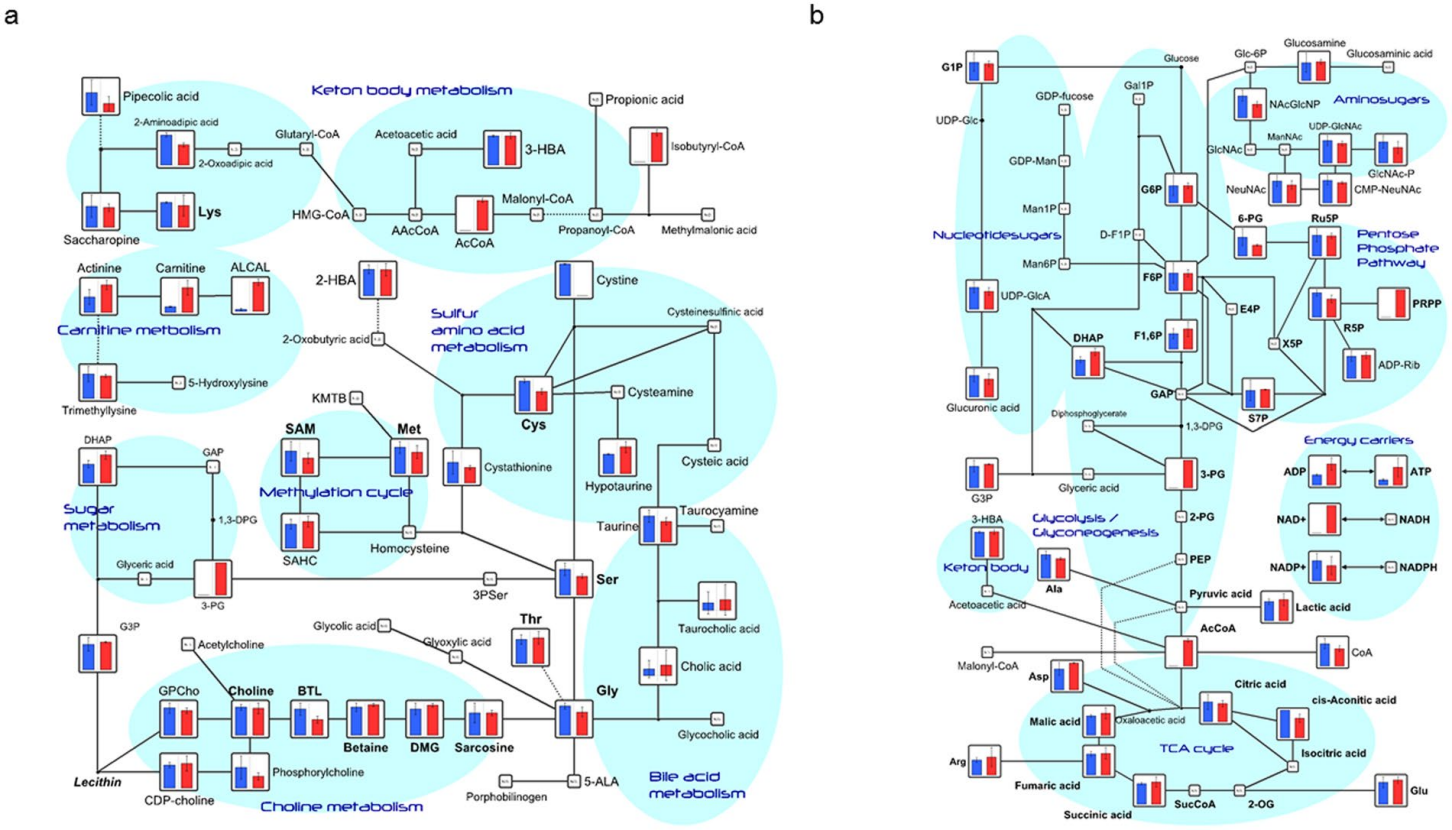
Comparison of metabolites identified by CE-TOF-MS. (**a**) The metabolites are superimposed on a metabolite pathway map involving the metabolism of L-carnitine and ketone bodies. (**b**) The metabolites are superimposed on a metabolite pathway map involving the metabolism of glycolysis, TCA cycle and energy carriers. Blue: HFD group. Red: HFD + L-carnitine group. The metabolites showing a significant difference between groups were indicated with *<0.05.

We performed a metabolomic analysis of the medaka livers from the HFD group in the second week of L-carnitine administration. Compared to the control animals, those administered L-carnitine exhibited increases in L-carnitine metabolites such as L-carnitine (ratio: 4.4), butyrylcarnitine (ratio: 7.7), and O-acetylcarnitine (ratio: 14), and a decrease in the L-carnitine (LC)/acyl-L-carnitine (ALC) ratio (Fig. 2a, Table 1). Although statistical significance was not calculated since values were below detection limits in the control group, acetyl-CoA and ATP increased upon L-carnitine treatment, which indicates β oxidation of fatty acids. The metabolites associated with glycolysis, tricarboxylic acid cycle (TCA cycle), and urea cycle, as well as amino acids and coenzymes did not exhibit clear changes indicating activated metabolic pathways (Fig. 2b). On the other hand, statistically significant increases were observed in L-carnitine treated medakas in spermidine levels (ratio: 1.3), γ-aminobutyric acid (GABA) (ratio: 1.6), allantoic acid (ratio: 2.0), and dihydroxyacetone phosphate (ratio: 1.5), while 2-aminoadipic acid (ratio: 0.7), 3-methylhistidine (ratio: 0.6), γ-Glu-Cys (ratio: 0.7), and 5-oxoproline (ratio: 0.7) decreased (Table 1).

**Table 1:** The metabolites showing a significant difference in levels between the HFD group and the HFD + L-carnitine group in LC-TOF-MS analysis.

KEGG number	compound name	control 1	control 2	control 3	carinitine 1	carinitine 2	carinitine 3	ratio	*p*-value
C_0130	*O*-Acetylcarnitine	3.2E-03	2.5E-03	7.4E-03	6.2E-02	5.5E-02	6.8E-02	14	0.0001
C_0144	Butyrylcarnitine	2.4E-04	3.2E-04	3.1E-04	2.3E-03	2.0E-03	2.5E-03	7.7	0.0001
C_0098	Carnitine	7.5E-03	8.6E-03	8.8E-03	3.6E-02	2.6E-02	4.8E-02	4.4	0.0117
C_0135	XC0061	3.5E-04	2.1E-04	2.8E-04	8.7E-04	5.3E-04	7.7E-04	2.6	0.0151
A_0098	ADP	5.5E-03	6.6E-03	6.3E-03	9.6E-03	1.7E-02	1.2E-02	2.1	0.0389
A_0043	Allantoic acid	9.7E-04	1.4E-03	1.5E-03	2.2E-03	2.4E-03	3.3E-03	2.0	0.0248
C_0017	GABA	2.9E-02	3.4E-02	2.2E-02	3.8E-02	4.4E-02	5.1E-02	1.6	0.0378
A_0034	Dihydroxyacetone phosphate	4.5E-03	4.0E-03	3.1E-03	5.5E-03	5.0E-03	6.7E-03	1.5	0.0475
C_0080	Spermidine	1.6E-03	1.4E-03	1.2E-03	1.8E-03	1.8E-03	2.0E-03	1.3	0.0208
A_0057	Pantothenic acid	7.4E-04	8.1E-04	6.3E-04	9.4E-04	9.3E-04	9.1E-04	1.3	0.0219
A_0014	5-Oxoproline	4.7E-04	5.9E-04	5.7E-04	4.6E-04	3.9E-04	3.5E-04	0.7	0.0443
C_0156	γ-Glu-Cys	1.9E-04	2.3E-04	2.6E-04	1.7E-04	1.4E-04	1.4E-04	0.7	0.0281
C_0097	2-Aminoadipic acid	2.6E-03	3.0E-03	2.8E-03	2.0E-03	2.0E-03	1.7E-03	0.7	0.0035
C_0105	3-Methylhistidine	2.9E-03	2.0E-03	2.5E-03	1.6E-03	1.8E-03	1.1E-03	0.6	0.0410
A_0108	ATP	8.9E-04	N.D.	6.4E-04	1.6E-03	4.2E-03	2.1E-03	3.4	
A_0093	Acetyl CoA_divalent	N.D.	N.D.	N.D.	9.7E-04	8.5E-04	8.9E-04	1<	
C_0042	Cys	1.5E-04	1.4E-04	1.6E-04	N.D.	8.8E-05	1.1E-04	0.6	
C_0039	2-Methylserine	1.3E-04	1.3E-04	N.D.	7.6E-05	9.7E-05	9.5E-05	0.7	
C_0143	Isobutyrylcarnitine	5.2E-05	N.D.	1.0E-04	1.9E-04	2.9E-04	2.9E-04	3.3	

Values represent the relative area of the detected peak for each metabolite. Ratio: the value in the HFD + L-carnitine group/the value in the HFD group. N.D.: not detected. The metabolites including “N.D.” are collectively shown in the bottom and their p-values are not presented.

### Comparison of lipid metabolism

**Figure 3:**
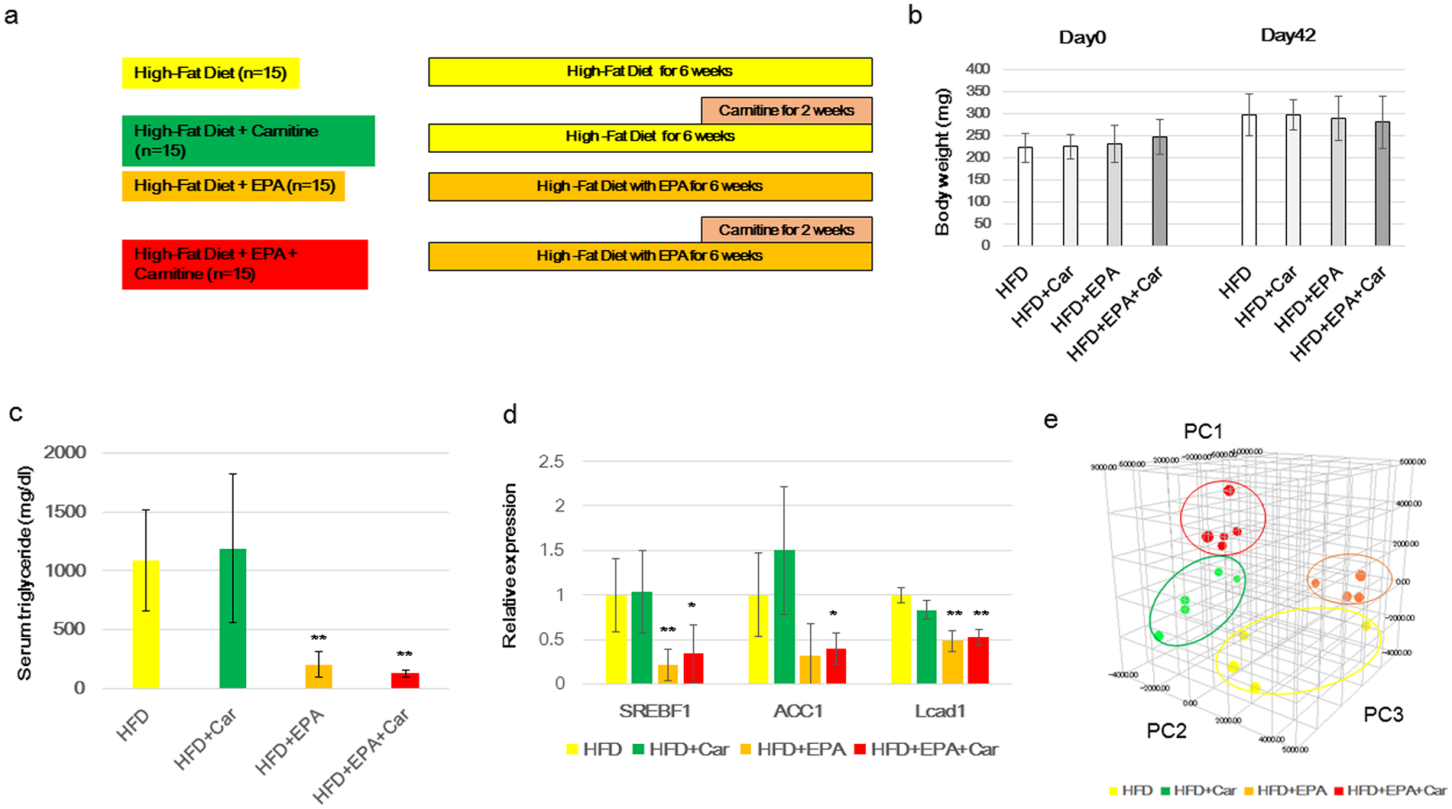
GC-MS analysis of changes in lipid metabolism. (**a**) A schematic diagram of the methods of feeding and agent administration. Medakas (n = 15 in a tank) were fed HFD or HFD + EPA (HFD + 5 wt.% EPA) and raised for 4 weeks. Following this, 1 mM L-carnitine was administered and they were raised for 2 weeks while the water was replaced every 3 days. At the end of the period, they were sacrificed. (**b**) Change in body weight due to L-carnitine administration. There was no clear difference in body weight among the groups before L-carnitine administration. On Day 42, the body weight significantly increased in the normal diet group and HFD group, but there was no difference between the HFD group and HFD + L-carnitine group. *Represents p < 0.05. (**c**) Comparison of serum triglyceride level. **Represents p < 0.01 compared to HFD group. (**d**) Changes in gene expression of Srebf1, Acc1, and Lcad in the liver. Data are means ± SD. *Represents p < 0.05, **Represents p < 0.01 compared to HFD group. (**e**) Principal component analysis normalized fatty acid data obtained from medaka livers. Percentage values indicated on the axes represent the contribution rate of the first (PC1), second (PC2), and third (PC3). Every group was clearly separated.

Since lipids could not be analysed in detail by capillary electrophoresis-time-of-flight/mass spectrometry (CE-TOF-MS), we performed gas chromatography-mass spectrometry (GC-MS) to analyse lipid molecular changes. We also analysed the effects of concomitant administration of L-carnitine and EPA. We also evaluated the effect of EPA on body weight by titrating EPA. After 2 months feeding of EPA, the body weight was decreased in 10% and 20% EPA administration groups (Supplementary Fig. S1). We examined the change in liver by HE staining and found that fat deposition in liver increased in all EPA administration groups. Medakas were divided into the HFD group, HFD + L-carnitine group, HFD + EPA group, and the HFD + EPA + carnitine group (number of fish was 15 in each group) to analyse changes in lipid metabolism in each of them (Fig. 3a). No significant changes in body weight were observed among the four groups (Fig. 3b). We also evaluated the triglyceride level in serum, and found that triglyceride level was greatly decreased in EPA and EPA + L-carnitine group (Fig. 3c). Furthermore we examined the mRNA expression level of fat related genes including sterol regulatory element-binding factor 1 (Srebf1), Acetyl-CoA carboxylase (Acc1), and long-chain acyl-CoA dehydrogenase (Lcad). Expressional level of these genes were decreased in EPA and EPA + L-carnitine group (Fig. 3d). We then evaluated fatty acid composition by GC-MS and separated the four groups by principal component analysis (PCA) (Fig. 3e). The lipid profiling of the HFD + L-carnitine group when compared to that of the HFD group showed significant decreases in pentadecanoate (15:0), margarate (17:0), linoleate (18:6n-6), ganma-linolenate (18:3n-6), and arachidonate (20:4n-6) (Table 2). In contrast, the comparison of the lipid profiles between the HFD group and the HFD + EPA group showed significant increases in EPA, Docosahexaenoic acid (22:6n-3) (DHA), and docosapentaenoate (DPA) (22:5n-3), and significant decreases in stearate and arachidonate (20:4n-6) in the latter group (Table 3). Comparative analysis of lipid metabolism between the HFD + EPA + L-carnitine group and the HFD group showed a significant increase in EPA (EPA was below detection limits in the HFD group), DHA and DPA and a significant decrease in arachidonate in the former group (20:4n-6) (Table 4). Other fatty acids, which were found to be decreased by L-carnitine administration, were not decreased by the concomitant administration of L-carnitine and EPA.

**Table 2:** The comparison of fatty acid profiles of the HFD group and HFD + L-carnitine group.

Annotation	Average HFD	Average HFD Car	Ratio (HFD + Car/HFD)	p*-value*
myristate;14:0	39864	34457	0.86	0.464
pentadecanoate;15:0	12806	9373	*0.73*	0.015
palmitoleate;(Z)16:1n-7	40622	31542	0.78	0.144
palmitate;16:0	938375	853913	0.91	0.363
margarate;17:0	19084	13799	***0.72***	**0.006**
ganma-linolenate;(Z)18:3n-6	20128	8200	*0.41*	0.029
linoleate;(Z)18:2n-6	129876	71945	*0.55*	0.037
petroselate;(Z)18:1n-12	2211886	1788602	0.81	0.078
oleate;(Z)18:1n-9	454157	360815	0.79	0.068
elaidate;(E)18:1n-9	38177	34025	0.89	0.324
cis-12-Octadecenoate;(Z)18:1n-6	38177	34025	0.89	0.324
stearate;18:0	318521	311843	0.98	0.601
nonadecanoate;19:0	12130	11679	0.96	0.222
arachidonate;(Z)20:4n-6	28383	21411	*0.75*	0.032
cis-5,8,11,14,17-Eicosapentaenoate;(Z)20:5n-3	n.a.	n.a.	n.a.	n.a.
eicosa-8,11,14-trienoate;20:3n-6	7323	7053	0.96	0.691
cis-11,14-Icosadienoate;(Z)20:2n-6	2134	1943	0.91	0.130
cis-11-icosenoate;(Z)20:1n-9	58754	80721	1.37	0.041
arachisate;20:0	5012	5079	1.01	0.897
cis-4,7,10,13,16,19-Docosahexaenoate;(Z)22:6n-3	18472	18154	0.98	0.905
cis-7,10,13,16,19-docosapentaenoate;(Z)22:5n-3	7977	9712	1.22	0.260

The relative areas of the peaks are shown. The ratio represents the values for the HFD + L-carnitine group to the values for the HFD group. A significant difference (p < 0.05) or (p < 0.01) is shown by one underline or two underlines. The ratio column is indicated by font that is underlined (increased; p < 0.05), bold (increased; p < 0.01), italic and underlined (decreased; p < 0.05) and italic and bold (decreased; p < 0.01).

**Table 3:** The comparison of fatty acid profiles of the HFD group and HFD + EPA group.

Annotation	Average HFD	Average HFD + EPA	Ratio (HFD + EPA/HFD)	p-value
myristate;14:0	39864	34970	0.88	0.53807
pentadecanoate;15:0	12806	13974	1.09	0.53512
palmitoleate;(Z)16:1n-7	40622	39112	0.96	0.81455
palmitate;16:0	938375	853716	0.91	0.26272
margarate;17:0	19084	19255	1.01	0.93569
ganma-linolenate;(Z)18:3n-6	20128	21848	1.09	0.75504
linoleate;(Z)18:2n-6	129876	124029	0.95	0.78848
petroselate;(Z)18:1n-12	2211886	2037043	0.92	0.46729
oleate;(Z)18:1n-9	454157	420540	0.93	0.48662
elaidate;(E)18:1n-9	38177	35555	0.93	0.53645
cis-12-Octadecenoate;(Z)18:1n-6	38177	35555	0.93	0.53645
stearate;18:0	318521	286353	*0.90*	*0.03838*
nonadecanoate;19:0	12130	11822	0.97	0.35015
arachidonate;(Z)20:4n-6	28383	22023	*0.78*	0.01977
cis-5,8,11,14,17-Eicosapentaenoate;(Z)20:5n-3	n.a.	6140	n.a.	n.a.
eicosa-8,11,14-trienoate;20:3n-6	7323	4131	***0.56***	**0.00035**
cis-11,14-Icosadienoate;(Z)20:2n-6	2134	1616	*0.76*	0.02649
cis-11-icosenoate;(Z)20:1n-9	58754	42155	*0.72*	0.01358
arachisate;20:0	5012	4983	0.99	0.94864
cis-4,7,10,13,16,19-Docosahexaenoate;(Z)22:6n-3	18472	51708	**2.80**	**8.7E-05**
cis-7,10,13,16,19-docosapentaenoate;(Z)22:5n-3	7977	14485	**1.82**	**0.00157**

The relative areas of the peaks are shown. The ratio represents the values for the HFD + EPA group to the values for the HFD group. A significant difference (p < 0.05) or (p < 0.01) is shown by one underline or two underlines. The ratio column is indicated by font that is underlined (increased; p < 0.05), bold (increased; p < 0.01), italic and underlined (decreased; p < 0.05) and italic and bold (decreased; p < 0.01).

**Table 4:** Comparison of fatty acid profiles of the HFD group and HFD + EPA + L-carnitine group.

Annotation	Average HFD	Average HFD + Car + EPA	Ratio (HFD + Car + EPA/HFD)	p*-value*
myristate;14:0	39864	45495	1.14	0.3747
pentadecanoate;15:0	12806	12022	0.94	0.3970
palmitoleate;(Z)16:1n-7	40622	40597	1.00	0.9954
palmitate;16:0	938375	835335	0.89	0.0956
margarate;17:0	19084	16310	0.85	0.1440
ganma-linolenate;(Z)18:3n-6	20128	12878	0.64	0.0970
linoleate;(Z)18:2n-6	129876	120579	0.93	0.6941
petroselate;(Z)18:1n-12	2211886	2192572	0.99	0.9155
oleate;(Z)18:1n-9	454157	436289	0.96	0.6564
elaidate;(E)18:1n-9	38177	42735	1.12	0.0929
cis-12-Octadecenoate;(Z)18:1n-6	38177	42735	1.12	0.0929
stearate;18:0	318521	280405	0.88	0.1704
nonadecanoate;19:0	12130	11668	0.96	0.3738
arachidonate;(Z)20:4n-6	28383	17889	***0.63***	**0.0010**
cis-5,8,11,14,17-Eicosapentaenoate;(Z)20:5n-3	n.a.	2079	n.a.	n.a.
eicosa-8,11,14-trienoate;20:3n-6	7323	7257	0.99	0.9544
cis-11,14-Icosadienoate;(Z)20:2n-6	2134	2610	1.22	0.3270
cis-11-icosenoate;(Z)20:1n-9	58754	82056	1.40	0.0375
arachisate;20:0	5012	6328	1.26	0.2704
cis-4,7,10,13,16,19-Docosahexaenoate;(Z)22:6n-3	18472	48002	**2.60**	**0.0001**
cis-7,10,13,16,19-docosapentaenoate;(Z)22:5n-3	7977	16180	**2.03**	**0.0021**

The relative areas of the peaks are shown. The ratio represents the values for the HFD + EPA + L-carnitine group to the values for the HFD group. A significant difference (p < 0.05) or (p < 0.01) is shown by one underline or two underlines. The ratio column is indicated by font that is underlined (increased; p < 0.05), bold (increased; p < 0.01), italic and underlined (decreased; p < 0.05) and italic and bold (decreased; p < 0.01). n.d.: not detected, n.a.: not available.

## Discussion

The role of L-carnitine in weight reduction in humans and mouse models has been reported in a number of studies. In these, weight reduction was the result of a combination of L-carnitine administration and walking in overweight women^9^ or L-carnitine administration for 14 days in a model involving endurance training^10^. In humans, most studies do not show a positive effect on the weight reduction facilitated by L-carnitine. In the present study also, there was no clear weight change in the HFD + L-carnitine group when compared with the HFD group, following 2 weeks of L-carnitine administration.

The improvement of fatty liver in humans and mice has been reported in several studies. Malaguarnera *et al*. reported an L-carnitine-mediated improvement in glucose plasma levels, lipid profiles, and histological manifestation in patients with NASH^11^. Xia *et al*. reported that L-carnitine supplements improved the fatty liver in type 2 diabetic mice, and that the increase in fatty acid oxidation and decrease in the L-carnitine/Actyl- L-carnitine ratio are important^12^. With regard to fish models, there is a report showing that L-carnitine administration in catfish resulted in a change in the lipid metabolism that caused lipid deposition in the liver and muscles^13^. In the current study, tissue staining showed a decrease in lipid deposition in the liver via L-carnitine administration. It was thereby demonstrated that L-carnitine administration may be useful in the improvement of fatty liver. In addition, as was previously reported, we observed an increase in the expression of SOD2, which is known to be involved in the mitochondrial oxidative stress pathway^14^. The result supports the idea that a suppression of oxidative stress in the liver by L-carnitine prevent NASH development.

The metabolomic analysis, which was performed to assess the mechanisms of the improvement of fatty liver by L-carnitine in more detail, demonstrated a 4.4-fold increase in the amount of L-carnitine found in the liver following L-carnitine administration. A 1.7-fold increase in physiological L-carnitine levels was previously reported in a mouse model of NASH^15^. The reason for the greater increase in our study is presumably that excessive L-carnitine is excreted into the urine in mice, while in the medaka model, the compound was dissolved in the water in the tank, which allowed medakas to maintain higher L-carnitine levels. Increases in levels of the L-carnitine metabolites, butyrylcarnitine (ratio: 7.7) and O-acetylcarnitine (ratio: 14.0), and a decrease in the LC/ALC ratio were observed (Table 2). In addition, although statistical significance could not be calculated for values below detection limits, acetyl-CoA and ATP levels can be considered to have increased upon L-carnitine administration, which suggests that β oxidation of lipids was enhanced to produce acetyl-CoA, resulting in ATP production (Table 1). There was an increase in ADP (ratio: 2.0) as well, but the ratio of the increase was higher in ATP (ratio: 3.4). That is, ATP was elevated more than ADP, as can be seen from the ATP/ADP ratio, which suggests that an increase in the adenine nucleotide pool activates the production of more ATP. The increase in ATP production by L-carnitine has also been reported in a study showing that in human primary chondrocytes, ATP production increased at 24 hours following 1 mM L-carnitine administration.

Among other metabolites that exhibited significant changes in the metabolomic analysis, spermidine (ratio: 1.3), a polyamine having strong anti-oxidant activity, acts as an intrinsic free radical scavenger. Spermidine is synthesized from spermine by spermidine/spermine N1-acetyltransferase using acetyl-CoA as a substrate.

An increase in dihydroxyacetone phosphate (DHAP) (ratio: 1.5) was also observed. Glycerin is converted to DHAP via glycerol-3-phosphate and enters the glycolytic pathway to be metabolized. An increase in DHAP consequently suggests advancement of lipolysis^16^.

There was a decrease in 3-methylhistidine (ratio: 0.6). Blood level of this molecule is reported to be useful as an index of protein catabolism^17^. The result suggests that because lipids are used as an energy source, the requirement for energy from protein catabolism is less.

Acetylated L-carnitine crosses the blood-brain barrier via membrane receptors to enter the brain and be converted into GABA. In fact, a study reported that L-carnitine acted to decrease ammonia levels in patients with liver cirrhosis and improved feelings of fatigue^11^.

2-Aminoadipic acid (2-AAA) (ratio: 0.7), a degradation product of lysine that has not been fully characterized, is a potential modulator of glucose homeostasis in humans. 2-AAA is an important marker in assessment of risk for development of diabetes in humans. A study reported that individuals with 2-AAA levels in the top quartile have a 4-fold risk of developing diabetes compared to the rest of the population^18^. Therefore, it is interesting that 2-AAA was decreased in the group with L-carnitine administration, as it suggested a possible decrease in risk for diabetes.

In the liver, acyl-CoA, generated from free fatty acids, is transferred into the mitochondria via the carnitine shuttle and is converted to acetyl-CoA, which is metabolized in the TCA cycle. Ketone bodies are known to be generated from acetyl-CoA when a large amount of acyl-CoA remains unmetabolised following the TCA cycle. However, no increase in ketone bodies was observed following L-carnitine administration in the current study (Fig. 3a). This suggests efficient transport of acyl-CoA into the mitochondria and subsequent metabolism.

Furthermore, a fact that has attracted attention in recent years is that dietary L-carnitine appears to cause the acceleration of atherosclerosis, owing to its conversion in the body to trimethylamine (TMA) and trimethylamine-N-oxide (TMAO) by enteric bacteria^19^. In the current study, neither TMA nor TMAO was found at elevated levels in either the HFD or HFD + L-carnitine group. The fact that TMA and TMAO were detected in the normal diet group suggested that the amount of TMA or TMAO synthesized in our medaka model in response to L-carnitine administration was small.

In general, L-carnitine is not required for short- and medium-chain fatty acids to enter the mitochondrial matrix as described before, but is necessary for long-chain fatty acids to do so. In order to analyse lipid profiles in more detail, we performed lipid analysis by GC-MS. Although the changes were not significant, most of the fatty acids measured generally decreased in the L-carnitine group. Linoleate (18:6n-6), margarate (17:0), ganma-linolenate (18:3n-6), arachidonate (20:4n-6), and pentadecanoate (15:0) levels were significantly lower in the HFD + L-carnitine group compared to those in the HFD group. This acyl-chain length preference probably depends on the substrate specificity of acyl-coenzyme A synthetase enzymes (ACSs;EC 6.2.1.x), the “activators” of fatty acids, which form a thioester with CoA^20^. However, much is still unknown about this mechanism and the details remain to be investigated. Further study on the detailed relationship between chain length of fatty acids and β oxidation of fatty acids by L-carnitine may be undertaken.

Although n-3 polyunsaturated fatty acids such as EPA and DHA are known to have anti-inflammatory and anti-cancer activity, their detailed mechanisms of action have not been fully understood. EPA is converted by an elongation enzyme to DPA, which is converted to DHA by a desaturation enzyme. Upon stimulation of the cells, the polyunsaturated fatty acid arachidonate (20:4n-6) is released from the cell membrane by phospholipase A2. Subsequently, prostaglandin and thromboxane generated by cyclooxygenase as well as leukotrienes generated by lipoxygenase from arachidonate (20:4n-6) function as lipid mediators. Since EPA and DHA have been shown to decrease in case of L-carnitine administration in *Pagrus major* ^21^, we analysed the effects of the concomitant administration of L-carnitine and EPA. When EPA alone was administered, significant increases in fatty acids derived from EPA, DHA and DPA, and significant decreases in stearate and arachidonate (20:4n-6) were observed (Table 3). We also found that fat deposition in liver increased in each EPA groups (Supplemental Fig. S1), suggesting that most deposited fat is EPA derived fatty acid, and change in fatty acid composition is effective in improving fatty liver. There is a report showing that EPA alone increases mitochondrial fatty acid oxidation, while DHA increases peroxisomal fatty acid oxidation^22^. The results of the present study, however, showed that β oxidation of some fatty acids was not changed much by EPA administration, suggesting that EPA is less efficient in decreasing fatty acid levels than L-carnitine. In spite of this, decreases in omega-6 fatty acid levels were observed in our study, as previously reported, which support the usefulness of EPA administration. The analysis of lipid metabolism in case of concomitant administration of HFD, L-carnitine and EPA compared to that in case of HFD administration demonstrated a significant increase in EPA and DHA levels and a decrease in arachidonate levels (20:4n-6) (Table 4). Other fatty acids that were decreased by L-carnitine administration were not decreased by concomitant administration of L-carnitine and EPA. This data indicates that elevated EPA prevents β oxidation of other fatty acids.

A significant decrease in arachidonate levels (20:4n-6) may be the cause of anti-inflammatory effects. It may be that under L-carnitine administration, EPA was preferentially utilised for β oxidation over other fatty acids. The increase in omega 3 fatty acids, known to decrease liver fat based on reported clinical studies^23^, and decrease in arachidonate levels (20:4n-6) are the most important benefits of concomitant administration of EPA and L-carnitine. Only a few detailed studies on the effects of the concomitant administration of EPA and L-carnitine have been reported so far, but the important results from our study warrant future further investigation to assess potential human application. L-carnitine is a highly convenient agent, with no significant side effects in humans reported in clinical studies. Additionally, no side effects were observed in the medakas in our study. In conclusion, we evaluated the usefulness of L-carnitine using a medaka model of fatty liver. The current study showed that the administration of L-carnitine alone is effective in improving fatty liver, and that concomitant administration of L-carnitine and EPA elevates this effectiveness. The detailed metabolome-level effects of L-carnitine on lipid metabolism were analysed based on results obtained using a small fish, medaka, in the current study, and the findings are expected to be valuable in developing treatment for fatty liver-related diseases in the future.

## Methods

### Animals and reagents

An inbred medaka strain (Kyoto-Cab) was used in this study^24^. The protocol was approved by the Committee on the Ethics of Animal Experiments, University of Yamaguchi. All methods were performed in accordance with the relevant guidelines and regulations by the Committee on the Ethics of Animal Experiments, University of Yamaguchi. All surgery for medaka was performed under tricaine anaesthesia. All efforts were made to minimize their suffering during the course of this study. Medakas were sacrificed via overdose of anaesthesia. The proportions of protein, fat and carbohydrate, as well as the fatty acid compositions of the control HFD diets that were used in this study are elucidated in a previous report. The control diet (Hikari Crest) was purchased from Kyorin Co. Ltd, Hyogo, Japan. The HFD was purchased from CLEA Japan Inc., Tokyo, Japan. For EPA treatment, eicosapentaenoic acid (EPA, purity >99%; Mochida Pharmaceutical Co. Ltd, Tokyo, Japan) was mixed with HFD32 at a concentration of 5% by weight.

### Western blot analysis

Protein lysates were obtained by homogenizing tissues or cell pellets in sample buffer containing 62.5 mM Tris-HCl (pH 6.8), 4% SDS, 200 mM dithiothreitol (DTT), 10% glycerol, and 0.001% bromophenol blue using a tissue to buffer ratio of 1:10 (w/v), followed by boiling. Western blot analysis was performed using purified polyclonal anti-human SOD2 rabbit IgG. Antibodies against β-actin (Sigma) were purchased from the indicated suppliers.

### Histology

Euthanized fish were slit open from the anal vent to the gills, and the entire body was fixed with 4% paraformaldehyde in 0.1 M phosphate buffer (Muto, Tokyo, Japan). The liver was dissected, dehydrated in alcohol, and embedded in paraffin according to standard procedures. Serial sections (3 μm thick) were cut and stained with hematoxylin and eosin (H&E). Intracellular lipids were stained with Oil Red O to analyse fat accumulation in the liver.

### Blood analysis

Blood samples were collected as described^8^. Fish were kept on ice for 1–2 minutes and then bled by cutting a ventral portion of the tail fin. Blood was collected in a microcapillary tube and the volume measured. Blood samples were kept at room temperature for 1 hour before centrifugation at 1200 × g for 30 minutes at 4 °C.TG contents were analysed using Fujifilm drychem 3500 (Japan).

### Real-time RT-PCR

Livers were isolated and total RNA was extracted and purified using the RNeasy kit (Qiagen, Hilden, Germany). cDNAs were synthesized using purified RNA plus random hexamers and the Transcriptor First Strand cDNA synthesis kit (Roche, Indianapolis, IN). Quantitative real-time RT-PCR was performed as described, and primers sequences were written in the report^7^.

### Metabolome analysis

Approximately 50 mg of frozen tissue was plunged into 1500 µL of 50% acetonitrile/Milli-Q water containing internal standards (Solution ID: 304-1002, Human Metabolome Technologies, Inc., Tsuruoka, Japan) at 0 °C in order to inactivate the enzymes present in the cells. The tissue was homogenized thrice at 1500 rpm for 120 s using a tissue homogenizer (Micro Smash MS100R, Tomy Digital Biology Co., Ltd., Tokyo, Japan) following which the homogenate was centrifuged at 2300 × g and 4 °C for 5 min. Subsequently, 800 µL of upper aqueous layer was centrifugally filtered through a Millipore 5-kDa cutoff filter at 9100 × g and 4 °C for 120 min to remove proteins. The filtrate was centrifugally concentrated and re-suspended in 50 µL of Milli-Q water for CE-MS analysis. Metabolome measurements were carried out through a facility service at Human Metabolome Technologies Inc., Tsuruoka, Japan.

### GC-MS analysis

After methanol was added, samples were shredded and subjected to sonication by adding an internal standard (C19 methylester). Chloroform was added and the chloroform phase was collected. After samples were dried by blowing a stream of nitrogen gas, hexane and then methanol were added and samples were incubated for 120 min. After water was added, the hexane phase was collected to use in GC-MS. We used the SHIMADZU QP-2010 Ultra and GC-MS Metabolic Components Database Ver. 2 software. In the statistical analysis, we applied data alignment by Fragment Align using the Pirouette software to perform PCA. The datasets generated during and/or analysed during the current study are available from the corresponding author on reasonable request.

### Statistical Analysis

To determine statistical significance, Welsh’s two-factor t-tests were performed in ArrayStudio (Omicsoft) or “R” to compare protein-normalized data between experimental groups; P < 0.05 was considered significant.

## Electronic supplementary material

Supplementary Fig. S1
