# Supplementary material for: Evaluation of the effects of L-carnitine on medaka (Oryzias latipes) fatty liver
Source: Sci Rep. 2017 Jun 5;7:2749. doi: 10.1038/s41598-017-02924-5 (PMC5459862; doi:10.1038/s41598-017-02924-5)
Supplement: Supplementary file 1 — Supplementary Fig. S1 [file 41598_2017_2924_MOESM1_ESM.pdf]

Supplementary material:

Evaluation of the effects of L**-carnitine on medaka (*Oryzias latipes*) fatty liver**

Koichi Fujisawa, Taro Takami, Aya Matsuzaki, Toshihiko Matsumoto, Naoki Yamamoto, Shuji Terai, and Isao Sakaida

Fig. S1.

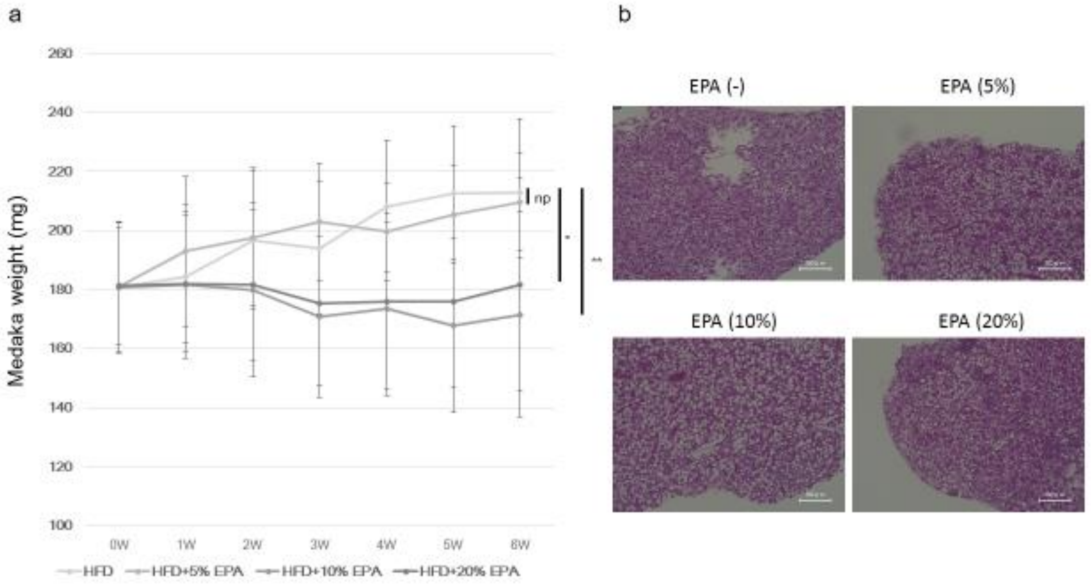

Fig. S1 Comparison of body weight and tissue staining after administration of EPA.

(a) Change in body weight following EPA administration. After 6 weeks feeding of EPA. * represents $p < 0.05$, ** represents $p < 0.01$, n.s represents not significant. (b) Staining of liver sections on week 6 (Haematoxylin and eosin staining).
